# Serological responses to COVID-19 Comirnaty booster vaccine, London, United Kingdom, September to December 2021

**DOI:** 10.2807/1560-7917.ES.2022.27.1.2101114

**Published:** 2022-01-06

**Authors:** Georgina Ireland, Heather Whitaker, Shamez N Ladhani, Frances Baawuah, Sathyvani Subbarao, Ezra Linley, Lenesha Warrener, Michelle O’Brien, Corinne Whillock, Oliver Martin, Paul Moss, Mary E Ramsay, Gayatri Amirthalingam, Kevin E Brown

**Affiliations:** 1 Immunisation and Vaccine Preventable Diseases Division, UK Health Security Agency, London, United Kingdom; 2 Statistics, Modelling and Economics Department, UK Health Security Agency, London, United Kingdom; 3 Brondesbury Medical Centre, Kilburn, London, United Kingdom; 4 Sero-Epidemiology Unit, UK Health Security Agency, Manchester, United Kingdom; 5 Virus Reference Department, UK Health Security Agency, London, United Kingdom; 6 Institute of Immunology and Immunotherapy, University of Birmingham, Edgbaston, United Kingdom; 7 Paediatric Infectious Diseases Research Group, St. George’s University of London, London, United Kingdom

**Keywords:** COVID-19, COVID-Vaccine, Antibody, Spike Protein, Immunity, Pfizer, AstraZeneca, Comirnaty, Vaxzevria

## Abstract

Serum samples were collected pre- and post-booster vaccination with Comirnaty in 626 participants (aged ≥ 50 years) who had received two Comirnaty doses < 30 days apart, two Comirnaty doses ≥ 30 days apart or two Vaxzevria doses ≥ 30 days apart. Irrespective of primary vaccine type or schedule, spike antibody GMTs peaked 2–4 weeks after second dose, fell significantly ≤ 38 weeks later and rose above primary immunisation GMTs 2–4 weeks post-booster. Higher post-booster responses were observed with a longer interval between primary immunisation and boosting.

In England, coronavirus disease (COVID-19) vaccine effectiveness (VE) against hospitalisation declined to 77.0% and 92.7% beyond 20 weeks post-vaccination, and to 78.7% and 90.4% against death, for the Vaxzevria vaccine (ChAdOx1-S, AstraZeneca, Cambridge, United Kingdom (UK)) and the Comirnaty vaccine (BNT162b2 mRNA, BioNTech-Pfizer, Mainz, Germany/New York, United States (US)), respectively. Greater waning of immunity was observed among older adults and those with underlying comorbidities [1] who were the first groups to be offered COVID-19 vaccination. In view of this and concerns about waning immunity, the emergence of the highly-transmissible Delta variant (Phylogenetic Assignment of Named Global Outbreak (Pango) lineage designation B.1.617.2) which causes more severe disease and can infect vaccinated individuals [2,3], high and sustained community infection rates in the UK and winter pressures on the national healthcare system, in September 2021 the UK Joint Committee on Vaccination and Immunisation recommended a third dose (booster) of a vaccine against COVID-19. The recommendation comprises either a single dose of Comirnaty vaccine or a half dose (50 µg) of Spikevax vaccine (mRNA-1273, Moderna, Cambridge, US) for selected population groups. Adults aged 50 years and older, individuals aged 16–49 years in clinical risk groups, adult carers and household contacts of immunosuppressed individuals and frontline health and social care workers are offered a booster dose at least 6 months after their second vaccine dose [4-6].

## COVID-19 vaccine responses after extended immunisation schedules

In England, the UK Health Security Agency initiated an evaluation of vaccine responses in adults aged 50 years and older who received the Comirnaty or Vaxzevria vaccines as part of the national immunisation programme to compare short vs longer interval vaccine schedules and monitor antibody waning over time [7]. The COVID-19 vaccine responses after extended immunisation schedules (CONSENUS) cohort has been described previously [7,8]. Immunocompetent adults aged 50 years and older in London were recruited in January 2021 to provide serial blood samples at 0, 3, 6, 9, 12, 15 and 20 weeks after their first dose of COVID-19 vaccine. As part of the national COVID-19 vaccine roll-out, participants received either (i) two Comirnaty doses < 30 days apart (Comirnaty-control); (ii) two Comirnaty doses ≥ 30 days apart (Comirnaty-extended); or (iii) two Vaxzevria doses ≥ 30 days apart (Vaxzevria-extended). Additional blood samples were taken before and after a Comirnaty booster dose. We describe the antibody kinetics after primary immunisation and booster vaccination in adults aged 50 years and older who were vaccinated as part of the national COVID-19 immunisation programme.

Serum samples were tested for nucleoprotein (N) antibodies as a marker of previous severe acute respiratory syndrome coronavirus 2 (SARS-CoV-2) infection (Elecsys Anti-SARS-CoV-2 total antibody assay, Roche Diagnostics, Basel, Switzerland). Results were expressed as a cut-off index (positive ≥ 1). Serum samples were also tested for spike (S) protein antibodies which could be infection- or vaccine-derived (Elecsys Anti-SARS-CoV-2 S total antibody assay, Roche Diagnostics). Results were expressed as arbitrary units (au)/mL (positive ≥ 0.8) to assess vaccine response. Individuals with ≥ 0.4 (au)/mL on the Roche N assay were considered to have had prior infection with SARS-CoV-2. This was assessed at enrolment. If participants tested N antibody positive after vaccination, indicating vaccine breakthrough, this and subsequent samples were removed from the analysis. The S antibody geometric mean titres (GMTs) were calculated with 95% confidence intervals (CI). Geometric mean ratios (GMR) of responses between time points were estimated using a mixed regression model on log responses including a random effect for each participant. Separate models were fitted for each vaccine group. The GMR of responses by vaccine type at each post-vaccination time point was estimated via regression on log Roche S responses and included age group and sex. Statistical analyses were performed using STATA version 14.2 (StataCorp, College Station, Texas, US).

## Antibody kinetics following primary COVID-19 vaccination

Of the 750 recruited participants, 626 provided serum samples for up to 38 weeks after their second vaccine dose (Table 1).

**Table 1:** Characteristics of participants with samples after second dose of the primary COVID-19 vaccination and after booster dose, London, United Kingdom, September–December 2021 (n = 626)^a^

Vaccine schedule	n	First and second dose schedule median in days (IQR)	Second and third dose (booster) schedule median in days (IQR)	Age median in years (IQR)	Sex				Ethnicity: white	
					Male		Female			
					n	%	n	%	n	%
All participants with samples provided after the second vaccine dose										
Vaxzevria-extended	240	72 (56–77)	NA	66 (55–71)	109	45	131	55	169	70
Comirnaty-extended	299	76 (73–77)		73 (70–78)	131	44	168	56	272	91
Comirnaty-control	87	21 (21–21)		76 (75–80)	42	48	45	52	78	90
All participants with samples provided after the third vaccine dose (booster)										
Vaxzevria-extended	50	56 (51–70)	186 (182–190)	69 (66–71)	20	–^b^	30	–^b^	45	–^b^
Comirnaty-extended	131	76 (74–76)	186 (182–188)	72 (69–75)	56	43	75	57	121	92
Comirnaty-control	52	21 (21–21)	262 (261–263)	77 (75–80)	27	–^b^	25	–^b^	48	–^b^

COVID-19: coronavirus disease; IQR: interquartile range; NA: not applicable.

^a^ All COVID-19 vaccine booster doses were Comirnaty.

^b^ Where n is less than 60, percentages have not been calculated.

For all three vaccine schedule groups, antibody GMTs peaked at 2–4 weeks after the second dose and then declined for all subsequent sampling points (Table 2) (Figure). Antibody GMTs declined by 68% at 36–38 weeks after the second dose for Comirnaty-control participants, by 85% at 24–29 weeks for Comirnaty-extended participants and by 78% at 24–29 weeks for Vaxzevria-extended participants. Antibody GMTs at 24–29 weeks remained higher in Comirnaty-extended participants than in Vaxzevria-extended participants (GMT 942; 95% CI: 797–1,113 vs 182; 95% CI: 124–268; p < 0.001). The latter value was similar to that of Comirnaty-control participants at 36–38 weeks (GMT 208; 95% CI: 150–289).

**Table 2:** Geometric mean responses and geometric mean response ratios of participants before and after second dose of the primary COVID-19 vaccination and after booster dose vaccination, London, United Kingdom, September–December 2021(n = 626)^a^

Vaccine schedule	Dose	Time sampled after dose (weeks)	n	Geometric mean responses		Within-individual geometric mean ratio of response relative to 2–4 weeks after second dose		Within-individual geometric mean ratio of response relative to 0–3 weeks before dose^b^	
				GMTs	95% CI	GMR	95% CI	GMR	95% CI
Vaxzevria-extended	First dose	< 3 pre second dose	147	29	23–36	NA		Ref.	
	Second dose	1	102	735	590–916	0.92	0.81–1.05	24.7	19.5–31.2
		2–4	126	812	650–1,015	Ref.		28.6	23.0–35.6
		5–8	86	615	493–767	0.73	0.64–0.84	NA	
		9–12	61	487	364–652	0.53	0.46–0.62		
		13–17	71	338	257–446	0.37	0.32–0.43		
		18–23	74	258	194–343	0.31	0.27–0.36		
		24–29	43	182	125–265	0.22	0.18–0.26		
	Third dose	< 3 pre third dose	29	189	131–273	NA		Ref.	
		2–4	43	10,799	8,510–13,704			57.2	38.8–84.2
Comirnaty- control	Second dose	2–4	80	694	540–893	Ref.		NA	
		18–23	72	330	261–418	0.49	0.4–0.6		
		36–38	56	208	150–289	0.32	0.25–0.39		
	Third dose	< 3 pre third dose	38	233	162–336	NA		Ref.	
		2–4	47	18,104	13,911–23,560			76.3	58.1–100.1
Comirnaty-extended	First dose	< 3 pre second dose	218	29	25–34	NA		Ref.	
	Second dose	1	142	7,44	6,021–9,198	1.31	0.21–1.43	272.6	234.4–4.317
		2–4	205	6,621	5,817–7,536	Ref.		217.8	190.9–248.6
		5–8	71	4,930	4,096–5,934	0.75	0.67–0.84	NA	
		9–12	106	2,698	2,323–3,135	0.43	0.39–0.47		
		13–17	173	1,770	1,545–2,029	0.29	0.27–0.32		
		18–23	43	1,344	1,081–1,670	0.2	0.17–0.23		
		24–29	126	942	797–1,113	0.15	0.13–0.16		
	Third dose	< 3	91	854	697–1,047	NA		Ref.	
		2–4	118	13,980	11,902–16,421			15.9	13.4–18.9
Individuals who were SARS-CoV-2-positive in the past									
Vaxzevria-extended	First dose	< 3 pre second dose	39	8,022	5,655–11,379	NA		Ref.	
	Second dose	1	25	9,138	5,997–13,924	1.19	0.94–1.51	1.1	0.9–1.3
		2–4	33	7,870	4,547–13,623	Ref.		1	0.9–1.2
		5–8	22	7,604	4,997–11,570	0.82	0.64–1.05	NA	
		9–12	12	6,667	3,549–12,526	0.71	0.52–0.97		
		13–17	19	4,895	2,949–8,125	0.63	0.49–0.82		
		18–23	15	6,830	4,021–11,601	0.51	0.38–0.68		
		24–29	5	2,878	1,367–6,062	0.49	0.33–0.75		
	Third dose	< 3 pre third dose	5	4,583	2,394–8,772	NA		Ref.	
		2–4	6	45,430	30,374–67,947			9.9	6.4–15.4
Comirnaty-control	Second dose	2–4	7	17,998	4,379–73,982	–^c^		NA	
		19–21	7	5,317	934–30,271				
		36–38	5	3,357	277–40,690				
	Third dose	< 3 pre third dose	3	–^c^		NA			
		2–4	4	–^c^					
Comirnaty- extended	First dose	< 3 pre second dose	28	1,797	768–47,025	NA		Ref.	
	Second dose	1	21	30,920	20,331–47,025	1.53	1.31–1.79	18.6	9.7–35.9
		2–4	25	24,680	15,900–38,308	Ref.		12.6	6.8–23.2
		5–8	7	11,975	5,327–26,917	0.6	0.48–0.76	NA	
		9–12	14	8,563	6,121–11,979	0.48	0.41–0.57		
		13–17	26	6,221	4,651–8,321	0.33	0.28–0.38		
		18–23	4	NA					
		24–29	20	4,185	2,944–5,949	0.2	0.17–0.23		
	Third dose	< 3 pre third dose	10	4,023	2,215–7,304	NA		Ref.	
		2–4	12	18,604	12,899–26,832			4.8	3.2–7.3
Convalescent sera, by weeks post symptom onset									
Unvaccinated people aged 50–89 years		3–7	141	55.3	39.4–77.7	NA			
		8–12	87	128.2	89.2–184.3				

CI: confidence interval; GMTs: geometric mean titres; GMR: geometric mean ratios; NA: not applicable; Ref: reference; SARS-CoV-2: severe acute respiratory syndrome coronavirus 2.

^a^ All COVID-19 vaccine booster doses were Comirnaty.

^b^ Column contains up to two different regression results per category, one for within-individual geometric mean ratio of response to the second dose, relative to 0–3 weeks before second dose, and another for within-individual geometric mean ratio of response to the booster dose, relative to 0–3 weeks before booster dose.

^c^ Geometric mean response was not calculated for categories with fewer than five individuals and within-individual geometric mean ratios were not calculated where categories had small numbers.

**Figure:**
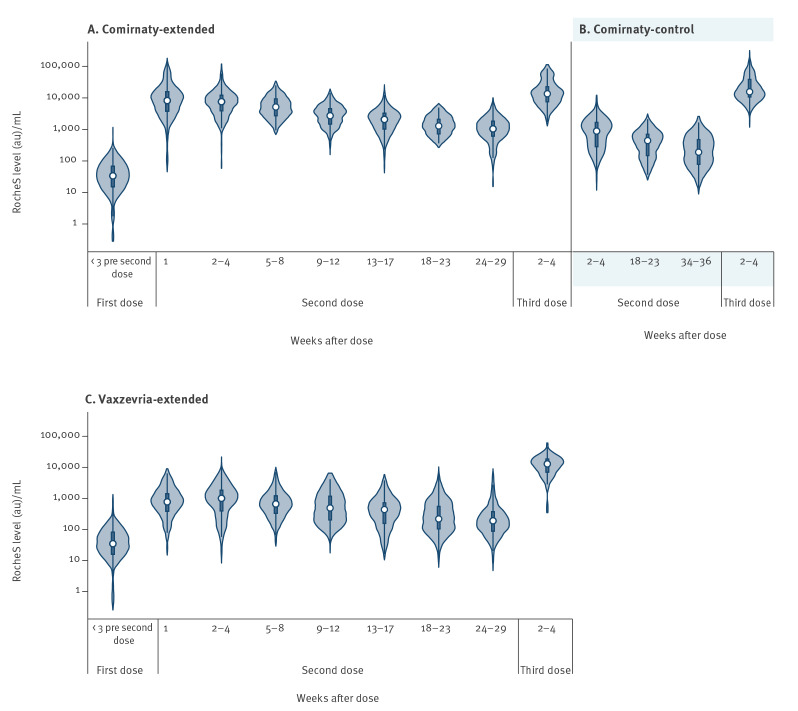
SARS-CoV-2 S antibody responses < 3 weeks before the second dose of the primary COVID-19 vaccination, after the second dose and after booster dose vaccination in previously uninfected individuals, London, United Kingdom, September–December 2021(n = 626)^a^

^a^ All COVID-19 vaccine booster doses were Comirnaty.

Regardless of primary vaccination type or schedule, antibody GMTs at all time points after the second dose were greater in previously infected participants. In Vaxzevria-extended participants, however, the decline in GMTs was smaller in previously infected individuals than in uninfected participants at 18–23 weeks (49% vs 68%). By comparison, a similar decline was observed for previously infected individuals compared with uninfected Comirnaty-extended participants at 24–29 weeks (80% vs 85%).

Two doses of Comirnaty or Vaxzevria vaccines provide high levels of protection against COVID-19, hospitalisation and death for at least 4–6 months after vaccination [8]. Unlike other countries that offered COVID-19 vaccination according to the 3–4-week interval as licensed by the UK Medicines and Healthcare Products Regulatory Agency, the UK recommended an extended 12-week schedule to expedite the rollout of the first dose of vaccine [8]. Subsequent studies have demonstrated higher antibody levels after the second dose with the extended schedule than with the licensed interval, potentially providing better longer-term protection [1,8]. However, antibody levels and clinical protection wane over time.

## Post-booster response

Serum samples were available for 51 Comirnaty-control participants, 130 Comirnaty-extended participants and 49 Vaxzevria-extended participants, 2–4 weeks after the Comirnaty booster. The boosted Vaxzevria-extended participants had a shorter interval between primary doses than all Vaxzevria-extended participants in the evaluation (median: 51 days vs 72 days), while Comirnaty-control participants had a longer interval between primary and booster doses (median: 262 days) than Comirnaty-extended or Vaxzevria-extended participants (median for both: 186 days).

Antibody GMTs at 2–4 weeks were highest in the Comirnaty-control participants (GMT 18,104; 95% CI: 13,911–23,560), followed by the Comirnaty-extended participants (GMT 13,98; 95% CI: 11,902–16,421) and Vaxzevria-extended participants (GMT 10,799; 95% CI: 8,510–13,704) (Table 2). Antibody GMTs in the Comirnaty-control participants were greater than for Vaxzevria-extended participants (p = 0.01). The largest post-booster increase in GMTs was in Comirnaty-control participants (76.3-fold increase), followed by Vaxzevria-extended (57.2-fold increase) and Comirnaty-extended (15.9-fold increase) participants (Table 2). Booster responses were not significantly affected by age (p < 0.05), but were higher in females (p = 0.008) compared to males. Sufficient post-booster information was available for previously-infected Vaxzevria-extended and Comirnaty-extended participants, where geometric mean responses increased 9.9-fold to 45,430 (95% CI: 30,374–67,947) and 4.9-fold to 18,604 (95% CI: 12,899–26,832) respectively.

### Ethical statement

The CONSENSUS study was approved by the Public Health England R&D Research Ethics and Governance Group (number: NR0253).

## Discussion

These early data show a rapid serological response to boosting with Comirnaty, with significantly higher antibody responses than those observed after the second dose. Serological assessments provide a measure of vaccine responses, but do not take into account innate or cellular immunity which also play an important role in protection [9,10]. However, S antibodies have been found to correlate well with neutralising antibodies [9,11,12]. Importantly, our cohort consists primarily of older adults who have a higher risk of severe COVID-19 and are, therefore, most likely to benefit from vaccination. Early data from Israel and England demonstrate substantially better protection against severe COVID-19, hospitalisation and death after booster vaccination [1,13], with similar data from many other countries [14]. In England, 14 days after boosting with Comirnaty among individuals aged 50 years and older, VE was similar in individuals who had received only primary doses of Comirnaty (87.4%; 95% CI: 84.9–89.4) and Vaxzevria (84.4%; 95% CI: 82.8–85.8) at least 140 days previously, although the analysis did not separate by schedule [1]. The higher post-booster GMTs observed in Comirnaty-control participants, who were the first to be vaccinated, could be because of the extended interval between primary and booster vaccines, which allowed more time for enhancing immune memory and greater waning of antibodies, both of which are likely to enhance post-booster responses. It could also be because of the difference in the primary course. However, the number of study participants is small, so results should be interpreted with caution. It is also likely that those who were vaccinated first are not representative of their age cohort.

## Conclusions

We observed high antibody responses following administration of a Comirnaty booster, irrespective of vaccine type or schedule used for primary immunisation. Our data suggest that a longer interval between primary immunisation and the booster may provide higher post-booster antibody responses and, potentially, longer lasting protection. Decisions on timing of booster doses should take into account the current and predicted epidemiological context to ensure that the most vulnerable groups are optimally protected during heightened periods of transmission. While the rates of severe disease, hospitalisations and deaths remain low in individuals receiving primary immunisation only, the booster programme will provide additional protection to those at highest risk of severe COVID-19 and help reduce infection rates across the population. Ongoing surveillance will be important for monitoring the duration of protection offered by booster doses and any need for additional doses in the future.

## Note

From 1 October 2021, the United Kingdom Health Security Agency (UKHSA) became fully operational. The UKHSA takes on the health protection responsibilities of Public Health England and incorporates National Health Service Test and Trace and the Joint Biosecurity Centre. The UKHSA is an executive agency of the Department of Health and Social Care. It is responsible for planning, preventing and responding to public health threats, and providing intellectual, scientific and operational leadership at national and local level, as well as on the global stage.
